# Multiple *Blastocystis* subtypes in Mediterranean marine turtles and cetaceans by amplicon-based NGS

**DOI:** 10.1016/j.fawpar.2025.e00307

**Published:** 2025-11-29

**Authors:** Marialetizia Palomba, Veronica Rodriguez-Fernandez, Renato Aco-Alburqueque, Meryam Carrus, Federica Marcer, Erica Marchiori, Mario Santoro, Tiziana Castrignanò, Daniele Canestrelli, Simonetta Mattiucci

**Affiliations:** a Department of Ecological and Biological Sciences, Tuscia University, Viale dell'Università, snc, 01100 Viterbo, Italy; b Department of Wellbeing, Health and Environmental Sustainability, "Sapienza University of Rome", Rome, Italy; c Department of Animal Medicine, Production and Health, University of Padova, Legnaro, Padua, Italy; d Department of Integrative Marine Ecology, Stazione Zoologica Anton Dohrn, Naples, Italy

**Keywords:** *Blastocystis*, Sea turtles, Cetaceans, Mediterranean Sea, NGS, Genetic diversity

## Abstract

*Blastocystis* is a genetically diverse enteric protist commonly found in humans and a wide range of vertebrate hosts. Although its prevalence and subtype (ST) distribution have been extensively studied in terrestrial ecosystems, its occurrence in marine organisms remains less known. In this study, we performed amplicon-based next-generation sequencing (NGS) to investigate, for the first time, the presence of *Blastocystis* in loggerhead sea turtles (*Caretta caretta*) and to expand existing data on ST diversity in cetaceans, stranded along the Italian Mediterranean coast. A total of 97 faecal samples were collected from 69 individuals of loggerhead sea turtles and 28 cetaceans. *Blastocystis* was detected in 44 % of the samples by real-time PCR—specifically in 39 loggerhead sea turtles and 4 fin whales (*Balaenoptera physalus*)—and further characterized by NGS. Ten STs were identified in loggerhead sea turtles and six in fin whales, with mixed infections frequently observed, particularly in turtles. Among the 18 STs detected, several represented new host records for marine organisms. ST4 was the most prevalent, especially in loggerhead sea turtles from the Tyrrhenian coast, and it exhibited a high degree of intra-subtype genetic variation. Comparison of ST4 sequences from this study with those of terrestrial origin revealed a certain level of substructuring; however, the most common haplotypes were shared between marine and terrestrial sources, supporting the hypothesis of a terrestrial origin for the marine STs. These findings highlight the potential use of *Blastocystis* STs occurring in marine megafauna as ecological indicators of faecal pollution from terrestrial origin in coastal marine environment. Moreover, they underscore the importance of applying a One Health framework, supported by NGS technologies, to elucidate the transmission dynamics of *Blastocystis* STs among humans, terrestrial, and marine hosts.

## Highlights

• First molecular survey of *Blastocystis* sp. in marine organisms by using NGS
• High relative proportion and genetic variation of zoonotic ST4.
• *Blastocystis* as a potential ecological indicator of terrestrial faecal contamination.

## 1 Introduction

*Blastocystis* is a ubiquitous enteric protist that colonizes the gastrointestinal tract of humans and a wide variety of vertebrates worldwide (Stensvold and Clark, 2016a; Stensvold and Clark, 2016a; Deng et al., 2021; Tsaousis et al., 2024; Stensvold, 2025). It is currently considered the most prevalent eukaryote in the human gut (Stensvold et al., 2020), yet its transmission routes, zoonotic potential, pathogenicity, and overall impact on host health remain only partially known (Sekar and Shanthi, 2013; Hublin et al., 2021; Stensvold and Clark, 2020; Stensvold et al., 2020).

Molecular studies have revealed remarkable genetic diversity within the genus. At least 48 subtypes (STs) have been identified to date based on SSU rDNA sequence polymorphisms, and this number is still increasing (Maloney et al., 2022; Oliwia Pawelec-Pęciak et al., 2025). Among subtypes, ST1 to ST4 are the most frequently detected in humans, although they have also been reported in various non-human hosts (Scanlan et al., 2015). Increasing evidence indicates that these STs may function as commensals rather than pathogens and can be associated with positive health outcomes (Deng et al., 2021, Deng et al., 2022; Tomiak and Stensvold, 2024; Deng and Tan, 2025a, Deng and Tan, 2025b). In contrast, several other STs appear to exhibit greater host specificity, being primarily associated with non-human animals (Jiménez et al., 2019; Nemati et al., 2021). In Italy, the occurrence of *Blastocystis* STs, as well as their intraspecific genetic diversity, has been extensively studied in terrestrial hosts, including domestic and wild animals as well as humans (Mattiucci et al., 2016; Gabrielli et al., 2021, Gabrielli et al., 2022; Guadano-Procesi et al., 2025). Despite substantial research in terrestrial ecosystems, the occurrence of *Blastocystis* in marine ecosystems has received little attention. However, in recent years, this interest has grown, particularly in relation to large marine vertebrates—such as cetaceans—which act as top predators or long-lived consumers occupying high trophic levels (Marangi et al., 2022; Muhammad Hafiz et al., 2024; Marangi and Boughattas, 2025; Palomba et al., 2023). In line with this emerging focus, several studies have reported the presence of *Blastocystis* STs in marine megafauna. The first pioneering large-scale study using end-point PCR, detected *Blastocystis* in stranded marine mammals—such as the common porpoise, sperm whale, and common seal— along the Northeast Atlantic coast, identifying four STs, including ST1–ST3 (Gantois et al., 2020). Using the same molecular approach, ST1 and ST3 were also detected in fin whale, sperm whale and long-finned pilot whale from the North-Western Mediterranean Sea (Marangi et al., 2021; Marangi and Boughattas, 2025). As for marine reptiles, a recent study reported the first detection of *Blastocystis* in this group, identifying ST8 in a captive green sea turtle (*Chelonia mydas*) in Malaysia, using end-point PCR (Muhammad Hafiz et al., 2024).

In recent years, high-throughput sequencing (NGS) technology has been widely used in terrestrial settings to detect mixed infections and uncover hidden diversity (e.g., Maloney et al., 2019; Maloney et al., 2022; Russini et al., 2020a, Russini et al., 2020b; Higuera et al., 2021). In contrast, to date, this approach has not been applied to investigate *Blastocystis* ST diversity in marine ecosystems.

In this context, the present study investigates the occurrence and genetic diversity of *Blastocystis* STs in wild loggerhead sea turtles and several cetacean species stranded along the Italian Mediterranean coastline. For the first time, an amplicon-based NGS approach was applied in the marine environment, in order to detect mixed infections and evaluate intra-subtype genetic variation of *Blastocystis* ST in these marine organisms.

## 2 Materials and methods

### 2.1 Sample collection and DNA extraction

**Fig. 1:**
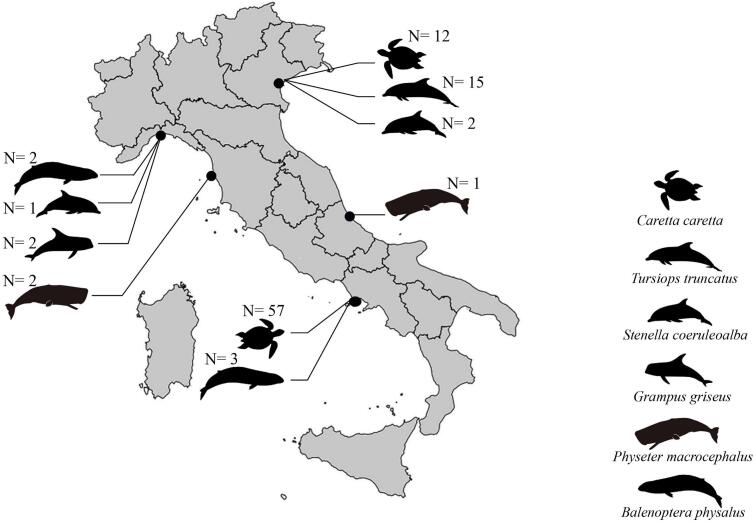
Map of the Italian Mediterranean coastline showing the stranding locations of cetacean species and loggerhead sea turtles from which faecal samples were collected between 2011 and 2021. The number of collected faecal samples from each host/locality, is also reported.

Faecal samples were collected from the rectum of fin whales (*Balaenoptera physalus*) (*N* = 5), sperm whales (*Physeter macrocephalus*) (*N* = 3), Risso's dolphins (*Grampus griseus*) (*N* = 2), striped dolphins (*Stenella coeruleoalba*) (N = 3), and *Tursiops truncatus* (*N* = 15), as well as from free-ranging loggerhead sea turtles *Caretta caretta* (*N* = 69). The individuals were dead stranded animals along the Italian Mediterranean coast between 2011 and 2021 (Fig. 1). Collected faecal samples were placed into sterile tubes and transferred in isothermal boxes to the Parasitology Laboratory of the Department of Public Health and Infectious Diseases, Sapienza University and held at −20 °C until DNA extraction. Extraction was performed from stool samples using the DNeasy Tissue Kit protocol (Qiagen, Valencia, CA), following the standard manufacturer's instruction.

### 2.2 Real-time PCR

As a first step, a molecular survey was performed using real-time PCR targeting a ∼ 300 bp fragment of the small subunit ribosomal DNA (SSU rDNA). The reaction was carried out in a total volume of 20 μL, consisting of 10 μL of SYBR Green PCR Master Mix (Applied Biosystems), 0.8 μL of each primer (10 μM)—BL18SPPF1 (5′-AGTAGTCATACGCTCGTCTCAAA-3′) and BL18SR2PP (5′-TCTTCGTTACCCGTTACTGC-3′) (Poirier et al., 2011)—and 2 μL of template DNA. The final volume was adjusted with nuclease-free distilled water. Details of the procedures followed Poirier et al. (2011).

### 2.3 Illumina MiSeq library preparation and sequencing

Library preparation and sequencing were outsourced to a commercial provider (IMR, Canada). The barcoding region of the small subunit (SSU) rDNA gene (V3 region) was amplified using primers modified with Illumina overhang adapter sequences Santín et al. (2011). This protocol followed Santín et al. (2011), with modifications for Illumina MiSeq compatibility as described by Maloney et al. (2019). Libraries were prepared using the Illumina 16S Metagenomic Sequencing Library Preparation protocol, including dual indexing to enable multiplexing. Libraries were quantified and pooled at a final concentration of 8 nM and sequenced using an Illumina MiSeq platform (2 × 300 bp paired-end reads) with 20 % PhiX as a sequencing control.

### 2.4 Bioinformatic analysis

Raw FASTQ files produced by Illumina MiSeq sequencing were processed using a bioinformatics pipeline. Quality control of raw reads was initially performed using FastQC (v. 0.12.1), which allowed assessment of base quality scores, GC content, sequence duplication levels, and adapter contamination. Adapter sequences, residual primers, and low-quality regions were removed using Trimmomatic (v. 0.39), with specific settings (ILLUMINACLIP:NexteraPE-PE.fa:2:30:10 SLIDINGWINDOW:4:15 MINLEN:36 HEADCROP:13) to detect and trim Nextera adapter sequences. After trimming, a second FastQC analysis was performed to confirm the quality improvement in the trimmed reads relative to the raw data. Paired-end reads were then merged using BBmerge (v. 39.06) by setting the k-mer “k = 31” and the parameter “extend2 = 50”. Only successfully merged reads were retained for downstream analysis (and unmerged discarded). Merged reads were subjected to quality filtering using the –fastq_maxee parameter in VSEARCH (v. 2.26.1). Following the protocol described by Maloney et al. (2019), we applied a threshold of 1.0 to prevent excessive read loss, ultimately retaining 1,740,811 high-quality reads. Dereplication was performed with vsearch (−-derep_fulllength), reducing the dataset to 603,896 unique reads. Chimeric sequences were detected and removed with vsearch (−-uchime_denovo), yielding 587,758 chimera-free reads. Reads were clustered into operational taxonomic units (OTUs) at 99 % sequence identity with vsearch (−-cluster_size). After clustering, OTUs shorter than 400 bp were discarded, resulting in a final set of 12,189 OTUs (OTUs mean length = 461 bp; mean number of OTUs per sample = 15).

To assign STs, all non-chimeric, dereplicated OTUs were queried against the *Blastocystis* PubMLST reference database (https://pubmlst.org/organisms/blastocystis-spp) using BLAST+ (v2.16.0) with the blastn algorithm. Only alignments with 100 % coverage and ≥ 99 % sequence identity were retained. STs were assigned on the basis of the top BLAST hit, following the consensus *Blastocystis* nomenclature (Stensvold and Clark, 2020).

All computationally intensive bioinformatic analyses were executed on a high-performance computing (HPC) platform (Castrignanò et al., 2020; Arcioni et al., 2024; Liberati et al., 2025a; Liberati et al., 2025b) to manage large-scale datasets (Lombardozzi et al., 2012).

All raw fastq files were deposited to the NCBI sequence read archive under the BioProject PRJNA1345741. The nucleotide sequences for unique OTUs obtained in this study were deposited in GenBank under the accession numbers PX506236-PX506245.

Intra-subtype genetic diversity was evaluated using standard diversity indices, including the number of haplotypes (Nh), nucleotide diversity (π), haplotype diversity (Hd), and the number of polymorphic sites (S), calculated with DnaSP v.5 (Librado and Rozas, 2009). *Fst* (fixation index) and *Nm* (gene flow) were also calculated using DnaSP v.5. The mean pairwise genetic distance, corrected using the Jukes–Cantor model, was computed using MEGA v.6 (Tamura et al., 2013). Phylogenetic analyses were inferred using Bayesian inference implemented in MrBayes v.3.2 (Ronquist et al., 2012), under the best-fitting nucleotide substitution model selected by jModelTest v.2.1 (Darriba et al., 2012). Haplotype networks were generated with Hapsolutely (Vences et al., 2024), applying the statistical parsimony method (95 % connection limit) as implemented in TCS.

## 3 Results

### 3.1 *Blastocystis* occurrence by real-time PCR

The occurrence of *Blastocystis* was detected by real-time PCR in 39 out of 69 faecal samples (*P* = 57.0 %) from loggerhead sea turtles. Specifically, in the Tyrrhenian coast, 36 of the 57 sea turtle samples (*P* = 63.0 %) were positive, compared with 3 out of 12 sea turtle samples (*P* = 25.0 %) from the Adriatic coast.

Among the 25 cetacean faecal samples analyzed, four (*P* = 16.0 %) were positive, all from fin whales. In detail, four out of five samples from fin whales (*P* = 80.0 %) were positive: all three individuals from the Tyrrhenian Sea (*P* = 100 %), and one out of two from the Ligurian Sea (*P* = 50.0 %) (Table 1). No *Blastocystis* was detected in any of the other cetacean species analyzed (Table 1).

**Table 1:** Occurrence and distribution of *Blastocystis* STs in the loggerhead sea turtles and cetacean species stranded along the Italian Mediterranean coast.

Host species	Location	N of samples	N of *Blastocystis* positive samples (P, %) by Real Time PCR and NGS	ST/s identified by NGS (% reads)
*Caretta caretta*				
	Tyrrhenian Sea	57	36 (63.1)	ST1 (1.3), ST3 (2.5), ST4 (54.1), ST6 (1.3), ST8 (2.6), ST10 (9.2), ST21 (5.1), ST23 (18.2), ST24 (3.2), ST27 (2.6)
	Adriatic Sea	12	3 (25.0)	ST4 (50), ST8 (16.6), ST10 (16.6), ST24 (16.6)
*Balenoptera physalus*				
	Tyrrhenian Sea	3	3 (100)	ST4 (33.3), ST6 (6.6), ST8 (6.6), ST10 (6.6), ST21 (6.6), ST23 (23.3), ST24, (16.6)
	Ligurian Sea	2	1 (50.0)	ST23 (100)
*Physeter macrocephalus*				
	Tyrrhenian Sea	2	–	–
	Adriatic Sea	1	–	–
*Stenella coeruleoalba*				
	Adriatic Sea	2	–	–
	Ligurian Sea	1	–	–
*Grampus griseus*				
	Ligurian Sea	2	–	–
*Tursiops truncatus*				
	Adriatic Sea	15	–	–

### 3.2 Host and spatial distribution of *Blastocystis* STs identified by NGS

A total of 5,430,481 paired-end sequencing reads were generated from the 43 *Blastocystis*-positive samples. After end trimming, merging, and quality filtering of read pairs, a total of 1,740,811 high-quality reads were retained with an average length of 461 bp. All sequences showed 99–100 % identity with *Blastocystis* reference sequences deposited in PubMLST (Public databases for molecular typing and microbial genome diversity) (Jolley et al., 2018). The dereplication and the removal of chimeric sequences resulted in 587,758 merged reads, which were used for OTU generation.

The overall prevalence of *Blastocystis*-positive individuals based on NGS data was consistent with that obtained by real-time PCR (Table 1).

**Fig. 2:**
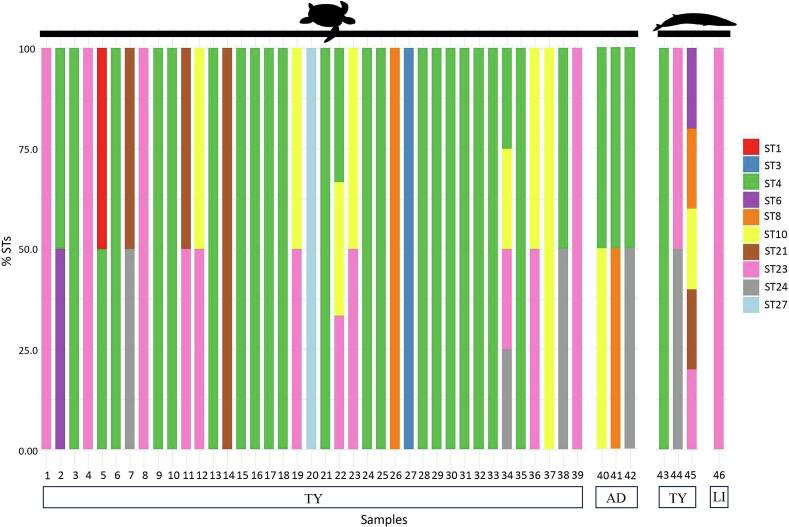
Relative proportions of *Blastocystis* STs detected in faecal samples from loggerhead sea turtles (samples 1–39) and fin whales (samples 40–43), based on amplicon-based NGS data. Each bar represents an individual sample, with colours indicating different STs. Sampling locations are indicated below the bars: TY = Tyrrhenian Sea, AD = Adriatic Sea, LI = Ligurian Sea.

**Fig. 3:**
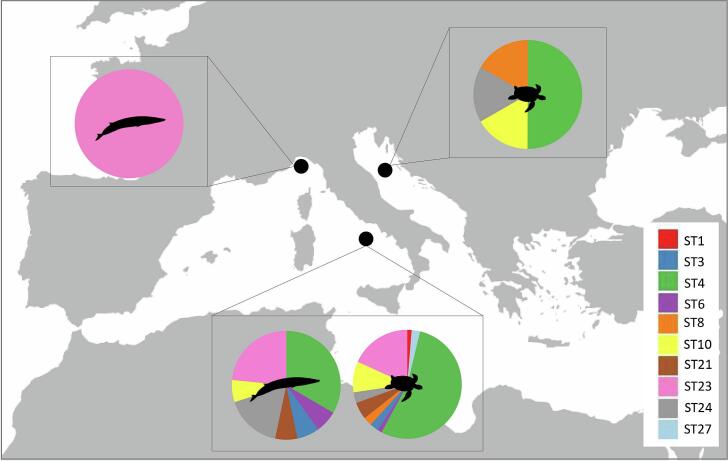
Distribution of *Blastocystis* STs in loggerhead sea turtles and fin whales from different areas of the Mediterranean coastline of Italy. Colours correspond to specific STs, as shown in the legend.

Using amplicon-based NGS, a total of 10 *Blastocystis* STs were identified in loggerhead sea turtles and 7 STs in fin whales (Table 1; Fig. 2). In loggerhead sea turtles from the Tyrrhenian coast, the following STs, with their relative proportion values, have been detected: ST1 (1.3 %), ST3 (2.5 %), ST4 (54.1 %), ST6 (1.3 %), ST8 (2.6 %), ST10 (9.2 %), ST21 (5.1 %), ST23 (18.2 %), ST24 (3.2 %), and ST27 (2.6 %) (Table 1; Fig. 3). In sea turtles from the Adriatic coast, the identified STs showed the following relative proportion ST4 (50.0 %), ST8 (16.6 %), ST10 (16.6 %), and ST24 (16.6 %) (Table 1; Fig. 3). In fin whales from the Tyrrhenian Sea, the detected STs included: ST4 (33.3 %), ST6 (6.6 %), ST8 (6.6 %), ST10 (6.6 %), ST21 (6.6 %), ST23 (23.3 %), and ST24 (16.6 %). In the single positive specimen from the Ligurian Sea, only ST23 was identified (Table 1; Fig. 3). Mixed infections involving two or more *Blastocystis* STs were detected in 16 samples: 14 from loggerhead sea turtles and 2 from fin whales.

Among the positive loggerhead sea turtle samples, 12 harboured two STs, while two contained more than two STs. In fin whales, one individual showed a mixed infection with two STs, while another one carried five distinct STs (Fig. 2, Fig. 3).

### 3.3 *Blastocystis* intra-subtype variation

At the intra-subtype level, a certain degree of genetic variation was observed among *Blastocystis* STs analyzed (Table 2). Specifically, the most represented ST in our dataset, i.e. ST4 (*N* = 32), exhibited the highest mean pairwise genetic distance, calculated by Jukes–Cantor model correction (JC = 0.0039). This ST has also shown a certain haplotype diversity (Hd = 0.756) with 11 haplotypes (Nh), 21 segregating sites and a moderate level of nucleotide diversity (π = 0.003). The ST23 (*N* = 23) showed similar nucleotide (π = 0.003) and haplotype diversity (Hd = 0.783), with 13 haplotypes (Nh) and 17 segregating sites. Likewise, ST24 (*N* = 14) displayed the same nucleotide diversity (π = 0.003), but a lower haplotype diversity value (Hd = 0.692), with only 7 haplotypes, 9 segregating sites and a moderate value of mean pairwise genetic distance (JC = 0.0033). Finally, ST10 (*N* = 15) and ST21 (*N* = 11) isolates showed the lowest nucleotide diversity values among all STs (Table 2). Finally, ST10 maintained a relatively high haplotype diversity (Hd = 0.790), with 6 haplotypes and 6 segregating sites. In contrast, ST21 displayed lower haplotype diversity (Hd = 0.345), with 3 haplotypes and 5 segregating sites. Due to limited sample sizes (N = 1–5), a reliable estimate of genetic diversity was not possible for ST1, ST3, ST6, ST8, and ST27.

**Table 2:** Genetic diversity values in *Blastocystis* STs detected in the present study. The table includes the number of sequences analyzed (N), mean pairwise genetic distance corrected using the Jukes–Cantor model (JC), nucleotide diversity per site (π), number of haplotypes (Nh), haplotype diversity (Hd), and number of segregating sites (S). Genetic diversity could not be estimated for ST1, ST3, ST6, ST8, and ST27 due to low sample sizes (N = 1–5).

Subtype (ST)	N	JC	π	Nh	Hd	S
ST4	32	0.0039	0.003	11	0.756	21
ST10	15	0.0029	0.002	6	0.790	6
ST21	11	0.0014	0.002	3	0.345	5
ST23	23	0.0035	0.003	13	0.783	17
ST24	14	0.0033	0.003	7	0.692	9

**Fig. 4:**
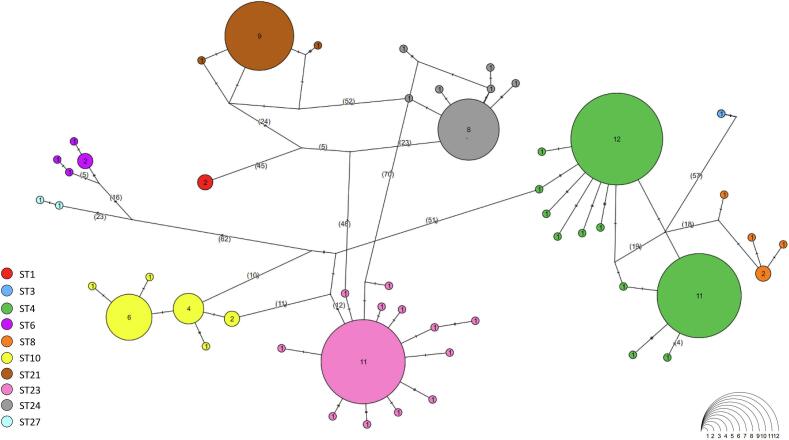
TCS network of the partial SSU rDNA sequences obtained from *Blastocystis* STs detected in the present study. Each haplogroup corresponds to a distinct ST, represented by a different colour. Circle sizes are proportional to the number of shared isolates per each haplotype.

**Fig. 5:**
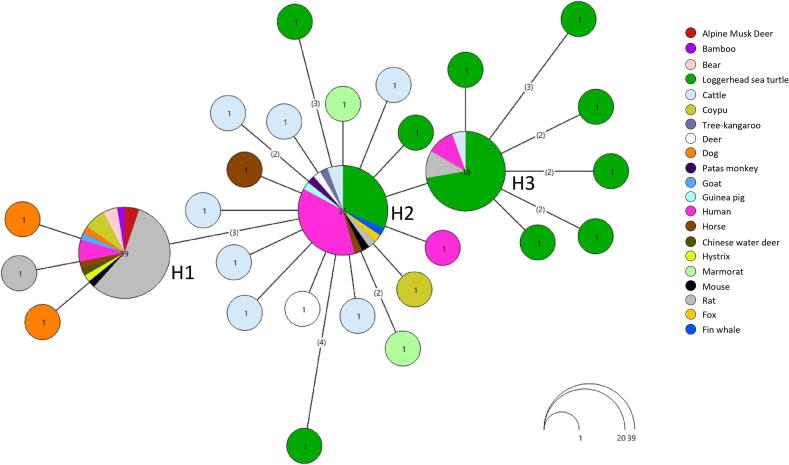
TCS network of partial SSU rDNA sequences of *Blastocystis* ST4 obtained in this study and from reference sequences available in NCBI. Each haplogroup corresponds to a distinct host, represented by a different colour. Circle size is proportional to the number of isolates sharing each haplotype.

**Supplementary Fig. 1:**
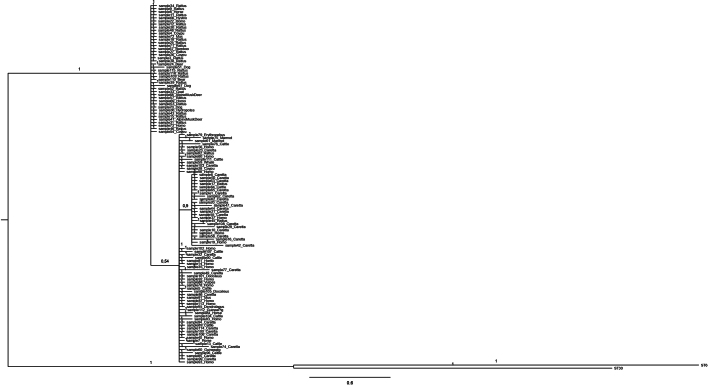
Phylogenetic tree from Bayesian inference on 18 s sequences of ST4 obtained in the present study, with respect to the sequences of ST4 at the same gene locus available in GenBank.

Observations on ST haplotype diversity were consistent with the network analysis (Fig. 4), which showed that ST23 and ST10 formed more distributed networks of interconnected haplotypes. Conversely, ST4 and ST3 appeared as compact clusters although they exhibited high haplotype richness and polymorphism. In particular, comparison of the ST4 sequences detected in this study with those previously deposited in GenBank revealed three main sequence clusters. The ST4 sequences obtained from loggerhead sea turtles were distributed across two major haplotypes (H3 and H2) (Fig. 5). One haplotype (H3; 18 sequences) was shared by rats, humans, and cattle, whereas the other (H2; 35 sequences) was mainly shared with humans and other terrestrial animals (Fig. 5). This clade also included the single ST4 haplotype detected from whale. In addition, several unique haplotypes exclusively associated with loggerhead sea turtles were observed (Fig. 5). The phylogenetic tree (Supplementary Fig. 1) supported this structure, showing the same clustering pattern among ST4 sequences. The analysis of genetic differentiation between terrestrial and marine ST4 sequences yielded a *Fst* value of ≈0.34 and a Nm value of ≈0.49.

## 4 Discussion

This study represents the first molecular survey of mixed infection by *Blastocystis* STs in marine organisms, by using an NGS platform. This approach enabled the detection of several mixed infection involving multiple *Blastocystis* STs in both the marine reptile and cetaceans stranded along the Mediterranean coastline. Our findings include the first record of *Blastocystis* STs in loggerhead sea turtles and provide novel insights into the host range and geographic distribution of this protist in cetaceans from the Mediterranean region. Notably, a markedly higher prevalence of *Blastocystis* was observed in sea turtles (57 %) compared to cetaceans (16 %), along with a greater diversity of STs in the former. In particular, the NGS approach here applied, has allowed to identify ST8 — the only one ST previously reported in a single green sea turtle with a prevalence of ∼7.7 % (1/13 individuals) (Muhammad Hafiz et al., 2024) — in co-infection with multiple other STs, namely ST1, ST3, ST4, ST6, ST10, ST21, ST23, ST24, and ST27. These results highlight a high diversity of *Blastocystis* STs in loggerhead turtles. The elevated prevalence and diversity of STs found in the present study may be explained by the foraging ecology of loggerhead sea turtles. Indeed, as benthic feeders inhabiting neritic coastal habitats, the loggerheads are routinely exposed to several anthropogenic influences, including urban and agricultural runoff that introduces faecal material from human and animal terrestrial sources into the marine environment (Serra et al., 2023). Two main transmission pathways of *Blastocystis* are plausible for loggerhead turtles: *(i)* ingestion of cyst-contaminated seawater and/or sediments; and *(ii)* trophic transfer through consumption of contaminated filter-feeding bivalve mollusks capable of retaining infecting cysts. Supporting the second hypothesized transmission route, *Blastocystis* ST3— also found in our samples— was recently detected in wild mussels (*Mytilus edulis*) from coastal France with an overall prevalence of 62 % (Ryckman et al., 2024), as well as in the Mediterranean mussels (*M. galloprovincialis*) from the Tyrrhenian coast with an overall prevalence of 10.3 % (Rodriguez-Fernandez et al., 2025), suggesting trophic exposure as a further plausible route of infection for this marine reptile in Mediterranean waters.

In addition, interestingly, although the number of individuals examined was low and does not allow to draw a conclusion, some variation in the relative proportion and composition of *Blastocystis* ST was observed between loggerhead sea turtles from the Tyrrhenian and Adriatic coasts. These results may reflect differences in foraging behaviour among loggerhead sea turtle populations (Mariani et al., 2023). Loggerhead sea turtles foraging in the Adriatic Sea seem to be predominantly of eastern Mediterranean origin and display strong seasonal fidelity to shallow feeding grounds dominated by crustaceans (Mariani et al., 2023). In contrast, Tyrrhenian individuals of loggerhead sea turtles, likely including both Mediterranean and Atlantic-origin turtles, have a broader habitat use and prey spectra—including mollusks and estuarine resources (Mariani et al., 2023). However, it is also worth noting that the stranded loggerhead sea turtles along the southeastern Tyrrhenian Sea represents the largest sample of the loggerhead sea turtles (*N* = 57) examined in the present study and they were sampled in the Gulf of Naples, a semi-enclosed basin of Italian coast. This densely populated area of southern Italian coast is subjected to significant industrial, port, urban, and agricultural pressures, all of which have a considerable impact on the water quality. This condition suggests that the loggerhead sea turtles, here examined, likely foraged in this contaminated environment, where the water may contain elevated concentrations of *Blastocystis* cysts from various sources. This phenomenon has been previously hypothesized for other protozoa, such as *Toxoplasma gondii*, in the same area (Santoro et al., 2020). Future eDNA analyses may help to confirm this hypothesis.

In our study, cetaceans showed a markedly lower prevalence of *Blastocystis* (16 %), with positive samples detected only in mysticetes—specifically, fin whales. Several STs were identified, including ST4, ST6, ST8, ST10, ST21, ST23, and ST24, in some cases occurring as mixed infections. None of the examined odontocetes (sperm whale, Risso's dolphin, striped dolphin, or bottlenose dolphin) resulted positive. Differences in feeding ecology—such as the filter-feeding behaviour of mysticetes versus the predatory habits of odontocetes—might influence exposure risk (Werth et al., 2024). However, this hypothesis remains speculative given the limited number of positive samples. Indeed, previous studies have reported *Blastocystis* in both groups, including odontocetes like common porpoises and sperm whales, with an overall prevalence of 9.2 % (3/22) (Gantois et al., 2020), as well as in fin whales and long-finned pilot whales, with an overall prevalence of 23.2 % (10/43) (Marangi et al., 2021; Marangi and Boughattas, 2025).

While ST1 and ST3 were exclusively detected in loggerhead sea turtles from the Tyrrhenian Sea, the remaining STs—ST4, ST6, ST8, ST10, ST21, ST23, and ST24—were found in both loggerhead sea turtles and fin whales. Among these, ST4 and ST8 are recognized as zoonotic and are commonly reported in industrialized countries (Gabrielli et al., 2022; Popruk et al., 2021), as well as frequently observed in human infections worldwide (Stensvold and Clark, 2016a; Barati et al., 2022), including Italy (Gabrielli et al., 2022; Guadano-Procesi et al., 2025).

In particular, the most frequent ST detected was ST4. It also showed a high degree of genetic variation (JC ≈ 0.004), which has not been previously reported. Although globally less common, ST4 is highly prevalent in Italy, where it accounts for nearly 20 % of human cases (Mattiucci et al., 2016; Gabrielli et al., 2021; Guadano-Procesi et al., 2025). Its epidemiology suggests a primary association with rodent reservoirs, though it has also been identified in wild birds, carnivores, and non-human primates (Stensvold and Clark, 2016b; Liu et al., 2024). The frequent detection of ST4 in our samples, particularly from Tyrrhenian stranding locations, may reflect land-sea transmission via runoff contaminated by terrestrial animal faeces. Consistently with this hypothesis, the most frequent ST4 haplotypes showed that sequences obtained from the marine animals investigated here were just those previously found in rats and humans. This pattern supports the idea of a primarily terrestrial origin of ST4 and suggests the existence of cross-environmental transmission routes. The presence of shared haplotypes between marine and terrestrial hosts indicates that sporadic transmission events across ecosystem boundaries may occur, maintaining a certain degree of genetic connectivity between isolates from the two environments. However, the moderate genetic substructuring observed between terrestrial and marine ST4 sequences (*Fst* ≈ 0.34), likely due to rare haplotypes so far distinctively observed in the two environments, could be due to the low number of isolates until now compared. However, on the other hand, it would also suggest that ecological or habitat-related barriers may limit the transmission between terrestrial and marine realm.

These findings suggest that *Blastocystis* may serve as a useful ecological indicator of terrestrial faecal contamination in marine environments. Its presence in marine hosts likely reflects input from land-based sources, highlighting its potential role as a sentinel organism for tracking land–sea pathogen transfer and environmental health.

## 5 Conclusions

This study represents the first application of an NGS approach to investigate *Blastocystis* STs in sea turtles, and it expands current knowledge on subtype diversity and intra-subtype variation in cetaceans from the Mediterranean Sea. The observed findings reveal a high prevalence and notable ST diversity of *Blastocystis* in loggerhead sea turtles, in contrast to the lower detection rates observed in cetaceans. The high occurrence of zoonotic STs and human-associated STs, along with the marine regional differences here observed, suggests that both anthropogenic and ecological factors contribute to shaping exposure risk of *Blastocystis* infection to these marine organisms.

These findings highlight the potential use of *Blastocystis* STs in marine megafauna as ecological indicators of environmental contamination in coastal marine environment.

Future research integrating *Blastocystis* STs occurrence into broader environmental monitoring strategies—particularly within a One Health framework—may offer valuable insights into the circulation of this pathogen/commensal in the marine realm.

The following are the supplementary data related to this article.

## CRediT authorship contribution statement

**Marialetizia Palomba:** Writing – review & editing, Writing – original draft, Methodology, Investigation, Formal analysis, Data curation, Conceptualization. **Veronica Rodriguez-Fernandez:** Writing – review & editing, Visualization. **Renato Aco-Alburqueque:** Methodology. **Meryam Carrus:** Visualization, Validation, Software. **Federica Marcer:** Writing – review & editing, Resources. **Erica Marchiori:** Writing – review & editing, Resources. **Mario Santoro:** Writing – review & editing, Resources. **Tiziana Castrignanò:** Writing – original draft, Supervision, Software, Formal analysis. **Daniele Canestrelli:** Supervision, Writing – review & editing. **Simonetta Mattiucci:** Writing – review & editing, Writing – original draft, Methodology, Investigation, Supervision, Project administration, Funding acquisition, Data curation, Conceptualization.

## Declaration of competing interest

The authors declare that they have no known competing financial interests or personal relationships that could have appeared to influence the work reported in this paper.
